# *In Silico* Analysis of Correlations between Protein Disorder and Post-Translational Modifications in Algae

**DOI:** 10.3390/ijms160819812

**Published:** 2015-08-20

**Authors:** Atsushi Kurotani, Tetsuya Sakurai

**Affiliations:** RIKEN Center for Sustainable Resource Science, 1-7-22 Suehiro-cho, Tsurumi-ku, Yokohama 230-0045, Japan; E-Mail: atsushi.kurotani@riken.jp

**Keywords:** algae, glycosylation, protein disorder, post-translational modifications, phosphorylation, ubiquitination

## Abstract

Recent proteome analyses have reported that intrinsically disordered regions (IDRs) of proteins play important roles in biological processes. In higher plants whose genomes have been sequenced, the correlation between IDRs and post-translational modifications (PTMs) has been reported. The genomes of various eukaryotic algae as common ancestors of plants have also been sequenced. However, no analysis of the relationship to protein properties such as structure and PTMs in algae has been reported. Here, we describe correlations between IDR content and the number of PTM sites for phosphorylation, glycosylation, and ubiquitination, and between IDR content and regions rich in proline, glutamic acid, serine, and threonine (PEST) and transmembrane helices in the sequences of 20 algae proteomes. Phosphorylation, O-glycosylation, ubiquitination, and PEST preferentially occurred in disordered regions. In contrast, transmembrane helices were favored in ordered regions. N-glycosylation tended to occur in ordered regions in most of the studied algae; however, it correlated positively with disordered protein content in diatoms. Additionally, we observed that disordered protein content and the number of PTM sites were significantly increased in the species-specific protein clusters compared to common protein clusters among the algae. Moreover, there were specific relationships between IDRs and PTMs among the algae from different groups.

## 1. Introduction

The intrinsically disordered regions (IDRs) of proteins have been extensively investigated over the past several years, and their numbers are significantly higher in eukaryotes than in bacteria or archaea [1,2]. More than one-third of eukaryotic proteins contain IDRs of more than 30 residues in length [3]. IDRs are characterized by a low abundance of order-promoting and bulky hydrophobic aromatic amino acids such as Ile, Leu, Val, Cys, Asn, Trp, Tyr, and Phe, a high abundance of disorder-promoting amino acids such as Ala, Arg, Gly, Gln, Ser, Glu, Lys and Pro, and a high net charge at neutral pH [1,4,5,6,7,8,9]. While IDRs form unstructured domains, predictions on which regions of a protein are disordered based on protein sequences can be used to select target regions for 3D structural analysis. For example, the disordered C-terminal 100 amino acid deletion mutant of the human nei-like DNA glycosylase (NEIL) protein was successfully expressed in *Escherichia coli*, purified, and crystallized [10]. More than 50 methods have been developed since the early 2000s for protein disorder predictions [6,11,12], including the development of structure-related databases and the design of IDR prediction algorithms.

IDRs often contain sites for various post-translational modifications (PTMs) such as phosphorylation, glycosylation, ubiquitination, acylation, and methylation, suggesting that PTMs favor the easily accessible and flexible disordered regions [13,14]. PTMs are involved in enzyme activity, protein localization, protein-protein interactions, signaling cascades, DNA repair, and cell division. PTMs commonly cause only small changes on the surface of the disordered region of a protein, whereas PTM-based protein-protein interactions often cause large-scale three-dimensional structural changes such as disorder-to-order transitions of IDRs [15]. Importantly, the larger structural changes are generally considered to have significant effects on protein functional diversity.

PTM prediction methods are well established. Several studies have reported that protein phosphorylation on Ser, Thr, and Tyr predominantly occurs in the disordered regions of animal proteins [16,17,18,19]. Proteome-wide analyses have also shown that there are correlations between IDRs and other PTMs such as glycosylation, acetylation, and methylation [20,21].

Proteins with IDRs have also been examined in plant proteomes [1,16,22]. For example, *Arabidopsis thaliana* (*A. thaliana*) proteins were found to be generally less disordered than human proteins. However, certain functional classes of *A. thaliana* proteins are significantly more enriched in disordered regions in response to their environment compared to human proteins [23]. This suggests that plants may use disorder independently as an easy and fast mechanism for introducing versatility into protein interaction networks that underlie complex biological processes to quickly and efficiently adapt to environmental changes of which they cannot escape [23]. Additionally, the relationships between PTMs and other cellular features are increasingly being explored in plant studies. For instance, phosphoproteomics and genetics analyses of *A. thaliana* have revealed insight into signaling pathways [24]. However, proteome information in many plant species is lacking. For example, *A. thaliana* is one of the most commonly studied plants and detailed genomic data are available. Conversely, one-third of the proteins in *A. thaliana* still lack functional annotations in terms of biological processes in the Gene Ontology database [24,25,26,27]. Recently, several large-scale experimental and computational approaches have been used to enhance our knowledge of plant species proteomes [27,28,29]. One such higher plant study was a proteome-wide computational analysis of the correlation between protein disorder and PTMs including Ser/Thr/Tyr-phosphorylation and O/N-linked protein glycosylation [19,21]. However, sufficient numbers of this type of study have not yet been performed, and plant researchers are currently developing better resources for omics analyses of plant species.

In order to enrich the gene annotation in plants and to understand gene and protein functions, we performed a proteome-wide correlation analysis of protein disorder with the major types of eukaryotic PTMs including Ser/Thr/Tyr-phosphorylation, O/N-linked glycosylation, and ubiquitination in 20 typical eukaryotic algae. We further analyzed sequences enriched in proline (P), glutamic acid (E), serine (S), and threonine (T) residues (PEST), as well as transmembrane helices. Phosphorylation, glycosylation, and ubiquitination were analyzed with a preference for occurrence in disordered regions using proteome sequence sets. We further investigated whether PEST regions and transmembrane helices preferentially occurred in disordered regions. In the process, it was revealed that protein disorder and multiple PTMs are favored by species-specific proteins among the algae.

## 2. Results

### 2.1. Comparison of Data Sets and Global Intrinsic Disorder in Algae Proteomes

**Figure 1:**
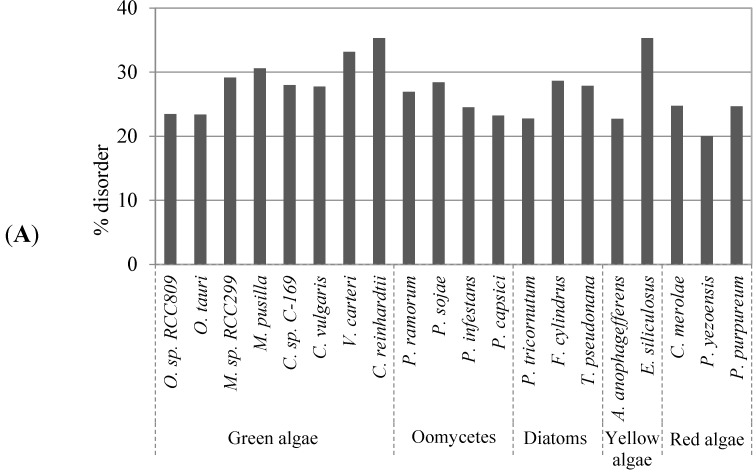

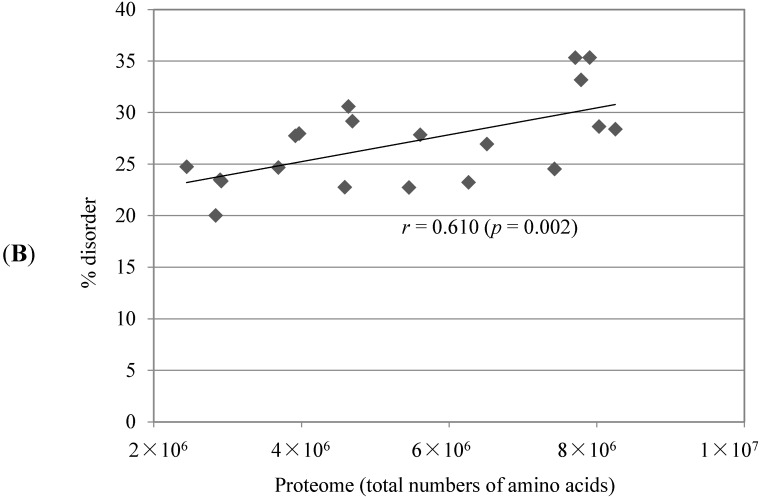
Evaluation of global protein disorder in algae proteomes. (**A**) Predicted disordered protein content in the studied algae proteomes computed with DISOPRED; (**B**) Results of a pairwise correlation analysis between the disorder content and the total number of amino acids in a proteome.

We prepared non-redundant sequence sets from 20 algae whole-protein sequences using the OrthoMCL tool [30] to remove sequences with >90% identity (for details, see Experimental Section 4.1). The number of sequences in the analyzed algae proteomes varied greatly from approximately 4800 in *Cyanidioschyzon merolae* to 20,000 in *Fragilariopsis cylindrus* (Table S1). Disordered protein content was determined using DISOPRED [31], which ranged from approximately 20% in *Pyropia yezoensis* to 35% in *Chlamydomonas reinhardtii* (Figure 1A). A similar trend was observed with another disorder prediction tool, RONN [32] (Figure S1). Additionally, we found that there was a correlation between disordered protein content and the total number of amino acids among the algae proteomes (Figure 1B).

### 2.2. Correlation between Intrinsic Disorder and Phosphorylation

Reversible protein phosphorylation, principally on serine, threonine, or tyrosine residues, is considered the most important PTM because of its role as a major regulatory mechanism in every living cell. Many studies have reported that protein phosphorylation sites are predominantly observed in the disordered regions of eukaryotic proteins, including those of plants [17,18,21]. Furthermore, the amino acid composition, sequence complexity, hydrophobicity, charge, and other sequence attributes of regions adjacent to phosphorylation sites are very similar to those of IDRs [18].

**Figure 2:**
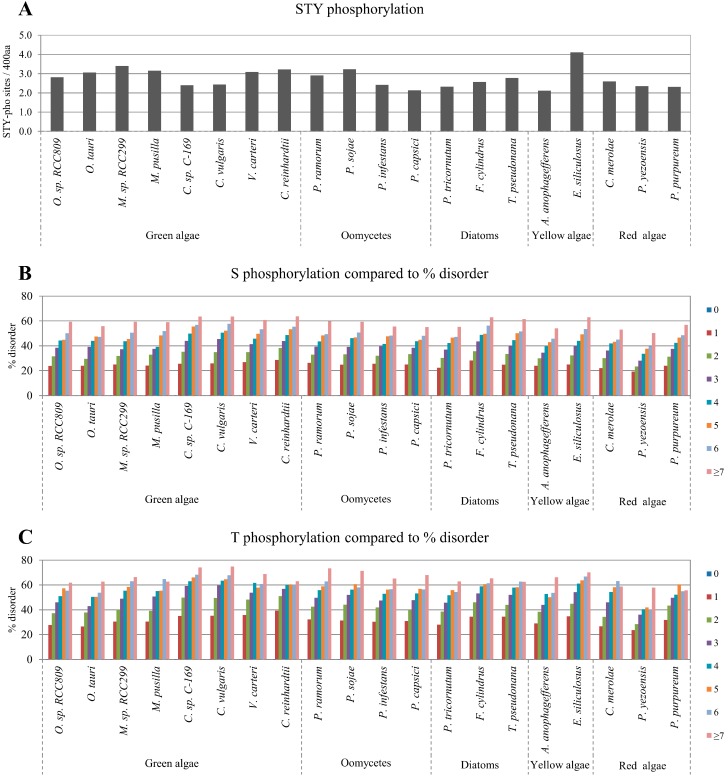

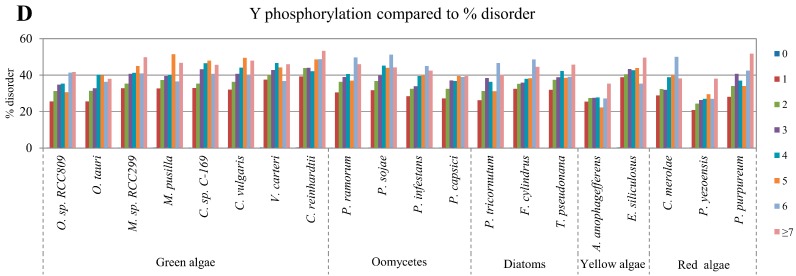
Correlations between disordered protein content and the number of phosphorylation sites. (**A**) Normalized predicted Ser (S), Thr (T) and Tyr (Y) phosphorylation sites per 400 amino acids in the studied algae proteomes; The relative percentage of disordered protein content with different numbers of predicted S, T and Y phosphorylation sites are presented in (**B**–**D**), respectively.

Recently, the bioinformatics tool Musite was developed for large-scale prediction of phosphorylation sites from protein sequences as a stand-alone application [17]. Therefore, the Musite tool was used to predict phosphorylation sites in the target algae proteomes. The number of predicted phosphorylation sites was normalized to the uniform length of 400 amino acids, rather than per sequence, to account for the difference in the average protein lengths in the datasets. Similarly, we used normalized values in the analyses of the number of glycosylation sites, ubiquitination sites, PEST regions, and transmembrane helices. No trend in the average abundance of phosphorylation sites in algae proteomes was found (Figure 2A). However, a strong positive correlation was observed between protein disorder and phosphorylation. For every algae proteome analyzed, there was a positive correlation between the predicted number (from 0 to ≥7) of Ser, Thr, or Tyr phosphorylation sites and disordered protein content (Figure 2B–D). The correlations between the number of Ser and Thr phosphorylation sites to disordered protein content were statistically significant as determined by one-tailed probability values and false discovery rates controlled by the Benjamini–Hochberg procedure [33] (Table 1). The positive correlation between disordered protein content and the number of phosphorylation sites was confirmed by using the alternative disorder prediction tool, RONN (Figure S3).

**Table 1:** Statistics of the correlation between protein disorder and proteomic parameters.

Species		S-pho	T-pho	Y-pho	O-gly	N-gly	PEST	Ubi	TM
Green algae	*O.* sp. RCC809	0.976	0.961	0.867	0.968	−0.705	0.953	0.993	−0.830
		2 × 10^−5^	3 × 10^−4^	3 × 10^−3^	4 × 10^−5^	3 × 10^−2^	2 × 10^−3^	4 × 10^−7^	5 × 10^−3^
		3 × 10^−5^	1 × 10^−3^	5 × 10^−3^	4 × 10^−5^	3 × 10^−2^	2 × 10^−3^	9 × 10^−7^	6 × 10^−3^
	*O. tauri*	0.967	0.958	0.895	0.973	−0.333	0.999	0.994	−0.803
		4 × 10^−5^	3 × 10^−4^	1 × 10^−3^	3 × 10^−5^	2 × 10^−1^	6 × 10^−7^	3 × 10^−7^	8 × 10^−3^
		4 × 10^−5^	1 × 10^−3^	3 × 10^−3^	3 × 10^−5^	2 × 10^−1^	1 × 10^−5^	7 × 10^−7^	9 × 10^−3^
	*M.* sp. RCC299	0.987	0.977	0.891	0.992	−0.869	0.982	0.992	−0.958
		3 × 10^−6^	8 × 10^−5^	1 × 10^−3^	5 × 10^−7^	3 × 10^−3^	2 × 10^−4^	8 × 10^−7^	9 × 10^−5^
		2 × 10^−5^	5 × 10^−4^	3 × 10^−3^	3 × 10^−6^	4 × 10^−3^	4 × 10^−4^	2 × 10^−6^	2 × 10^−4^
	*M. pusilla*	0.985	0.979	0.701	0.992	−0.970	0.994	0.990	−0.976
		4 × 10^−6^	6 × 10^−5^	3 × 10^−2^	7 × 10^−7^	3 × 10^−5^	3 × 10^−5^	1 × 10^−6^	2 × 10^−5^
		2 × 10^−5^	6 × 10^−4^	3 × 10^−2^	3 × 10^−6^	2 × 10^−4^	1 × 10^−4^	2 × 10^−6^	5 × 10^−5^
	*C.* sp. C-169	0.980	0.935	0.831	0.988	−0.980	0.970	0.968	−0.950
		1 × 10^−5^	1 × 10^−3^	5 × 10^−3^	2 × 10^−6^	1 × 10^−5^	7 × 10^−4^	4 × 10^−5^	2 × 10^−4^
		3 × 10^−5^	1 × 10^−3^	8 × 10^−3^	5 × 10^−6^	2 × 10^−4^	9 × 10^−4^	5 × 10^−5^	3 × 10^−4^
	*C. vulgaris*	0.974	0.929	0.846	0.986	−0.967	0.971	0.976	−0.966
		2 × 10^−5^	1 × 10^−3^	4 × 10^−3^	3 × 10^−6^	4 × 10^−5^	6 × 10^−4^	2 × 10^−5^	5 × 10^−5^
		3 × 10^−5^	1 × 10^−3^	6 × 10^−3^	7 × 10^−6^	2 × 10^−4^	9 × 10^−4^	2 × 10^−5^	1 × 10^−4^
	*V. carteri*	0.982	0.908	0.578	0.958	−0.883	0.965	0.910	−0.985
		7 × 10^−6^	2 × 10^−3^	7 × 10^−2^	9 × 10^−5^	2 × 10^−3^	9 × 10^−4^	8 × 10^−4^	4 × 10^−6^
		2 × 10^−5^	2 × 10^−3^	7 × 10^−2^	9 × 10^−5^	4 × 10^−3^	1 × 10^−3^	8 × 10^−4^	3 × 10^−5^
	*C. reinhardtii*	0.976	0.886	0.879	0.987	−0.805	0.962	0.967	−0.987
		2 × 10^−5^	4 × 10^−3^	2 × 10^−3^	2 × 10^−6^	8 × 10^−3^	1 × 10^−3^	4 × 10^−5^	3 × 10^−6^
		3 × 10^−5^	4 × 10^−3^	4 × 10^−3^	5 × 10^−6^	1 × 10^−2^	1 × 10^−3^	5 × 10^−5^	3 × 10^−5^
Oomycetes	*P. ramorum*	0.974	0.967	0.903	0.991	−0.971	0.995	0.995	−0.941
		2 × 10^−5^	2 × 10^−4^	1 × 10^−3^	1 × 10^−6^	3 × 10^−5^	2 × 10^−5^	1 × 10^−7^	2 × 10^−4^
		3 × 10^−5^	9 × 10^−4^	3 × 10^−3^	3 × 10^−6^	3 × 10^−4^	9 × 10^−5^	6 × 10^−7^	3 × 10^−4^
	*P. sojae*	0.975	0.932	0.976	0.985	−0.890	0.996	0.994	−0.948
		2 × 10^−5^	1 × 10^−3^	2 × 10^−5^	5 × 10^−6^	2 × 10^−3^	1 × 10^−5^	2 × 10^−7^	2 × 10^−4^
		3 × 10^−5^	1 × 10^−3^	2 × 10^−4^	8 × 10^−6^	3 × 10^−3^	6 × 10^−5^	7 × 10^−7^	3 × 10^−4^
	*P. infestans*	0.972	0.947	0.982	0.995	−0.533	0.984	0.996	−0.960
		3 × 10^−5^	6 × 10^−4^	8 × 10^−6^	1 × 10^−7^	9 × 10^−2^	2 × 10^−4^	7 × 10^−8^	8 × 10^−5^
		3 × 10^−5^	1 × 10^−3^	2 × 10^−4^	1 × 10^−6^	1 × 10^−1^	4 × 10^−4^	5 × 10^−7^	2 × 10^−4^
	*P. capsici*	0.971	0.953	0.923	0.990	−0.462	0.966	0.995	−0.943
		3 × 10^−5^	4 × 10^−4^	5 × 10^−4^	1 × 10^−6^	1 × 10^−1^	9 × 10^−4^	2 × 10^−7^	2 × 10^−4^
		3 × 10^−5^	1 × 10^−3^	2 × 10^−3^	3 × 10^−6^	1 × 10^−1^	1 × 10^−3^	7 × 10^−7^	3 × 10^−4^
Diatom	*P. tricornutum*	0.977	0.950	0.849	0.996	0.900	0.998	0.996	−0.935
		1 × 10^−5^	5 × 10^−4^	4 × 10^−3^	6 × 10^−8^	1 × 10^−3^	2 × 10^−6^	9 × 10^−8^	3 × 10^−4^
		3 × 10^−5^	1 × 10^−3^	6 × 10^−3^	1 × 10^−6^	3 × 10^−3^	2 × 10^−5^	4 × 10^−7^	4 × 10^−4^
	*F. cylindrus*	0.977	0.940	0.927	0.982	0.919	0.972	0.998	−0.977
		1 × 10^−5^	8 × 10^−4^	5 × 10^−4^	7 × 10^−6^	6 × 10^−4^	6 × 10^−4^	2 × 10^−8^	2 × 10^−5^
		3 × 10^−5^	1 × 10^−3^	2 × 10^−3^	1 × 10^−5^	2 × 10^−3^	1 × 10^−3^	4 × 10^−7^	6 × 10^−5^
	*T. pseudonana*	0.979	0.947	0.789	0.993	0.765	0.997	0.991	−0.981
		1 × 10^−5^	6 × 10^−4^	1 × 10^−2^	5 × 10^−7^	1 × 10^−2^	7 × 10^−6^	9 × 10^−7^	9 × 10^−6^
		3 × 10^−5^	1 × 10^−3^	1 × 10^−2^	3 × 10^−6^	2 × 10^−2^	5 × 10^−5^	2 × 10^−6^	4 × 10^−5^
Yellow algae	*A. anophagefferens*	0.975	0.937	0.386	0.979	−0.951	0.989	0.948	−0.991
		2 × 10^−5^	9 × 10^−4^	2 × 10^−1^	1 × 10^−5^	1 × 10^−4^	9 × 10^−5^	2 × 10^−4^	9 × 10^−7^
		3 × 10^−5^	1 × 10^−3^	2 × 10^−1^	2 × 10^−5^	6 × 10^−4^	2 × 10^−4^	2 × 10^−4^	2 × 10^−5^
	*E. siliculosus*	0.993	0.965	0.381	0.978	−0.875	0.991	0.982	−0.931
		5 × 10^−7^	2 × 10^−4^	2 × 10^−1^	1 × 10^−5^	2 × 10^−3^	6 × 10^−5^	7 × 10^−6^	4 × 10^−4^
		5 × 10^−6^	8 × 10^−4^	2 × 10^−1^	2 × 10^−5^	4 × 10^−3^	2 × 10^−4^	1 × 10^−5^	5 × 10^−4^
Red algae	*C. merolae*	0.971	0.989	0.965	0.968	−0.086	0.970	0.967	−0.936
		3 × 10^−5^	1 × 10^−5^	5 × 10^−5^	4 × 10^−5^	4 × 10^−1^	7 × 10^−4^	4 × 10^−5^	3 × 10^−4^
		3 × 10^−5^	3 × 10^−4^	3 × 10^−4^	4 × 10^−5^	4 × 10^−1^	9 × 10^−4^	5 × 10^−5^	4 × 10^−4^
	*P. yezoensis*	0.995	0.937	0.907	0.980	−0.942	0.948	0.947	−0.731
		1 × 10^−7^	9 × 10^−4^	9 × 10^−4^	1 × 10^−5^	2 × 10^−4^	2 × 10^−3^	2 × 10^−4^	2 × 10^−2^
		2 × 10^−6^	1 × 10^−3^	3 × 10^−3^	2 × 10^−5^	8 × 10^−4^	2 × 10^−3^	2 × 10^−4^	2 × 10^−2^
	*P. purpureum*	0.983	0.923	0.827	0.973	−0.378	0.984	0.997	−0.951
		6 × 10^−6^	1 × 10^−3^	6 × 10^−3^	2 × 10^−5^	2 × 10^−1^	2 × 10^−4^	2 × 10^−8^	1 × 10^−4^
		2 × 10^−5^	2 × 10^−3^	8 × 10^−3^	3 × 10^−5^	2 × 10^−1^	4 × 10^−4^	2 × 10^−7^	3 × 10^−4^

Pearson correlation coefficients, their associated *p* values, and false discovery rates (Benjamini–Hochberg procedure) are presented in the upper, middle and lower rows, respectively, for all analyzed correlations between IDRs and proteomic parameters. Shaded values indicate *p* values greater than 0.05 that were not considered statistically significant. The column names stand for S phosphorylation, T phosphorylation, Y phosphorylation, N-linked glycosylation, O-linked glycosylation, region enriched in proline (P), glutamic acid (E), serine (S), and threonine (T) residues (PEST), ubiquitination, and transmembrane helices.

### 2.3. Correlation between Intrinsic Disorder and Glycosylation

**Figure 3:**
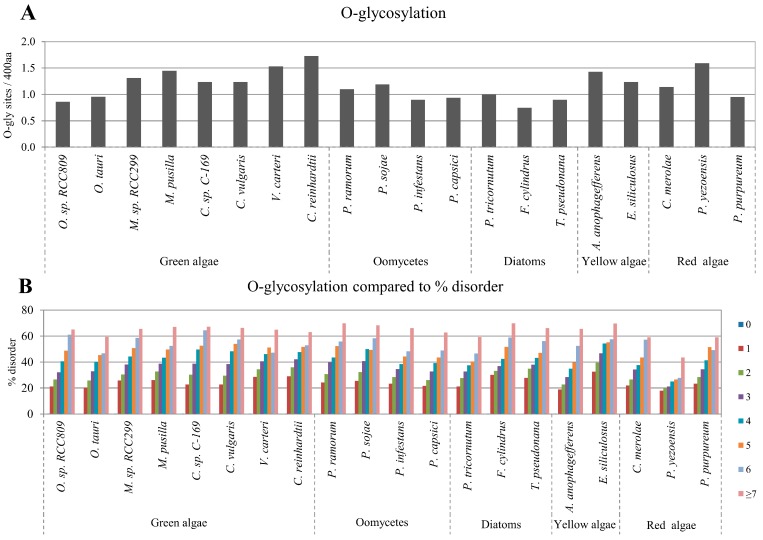

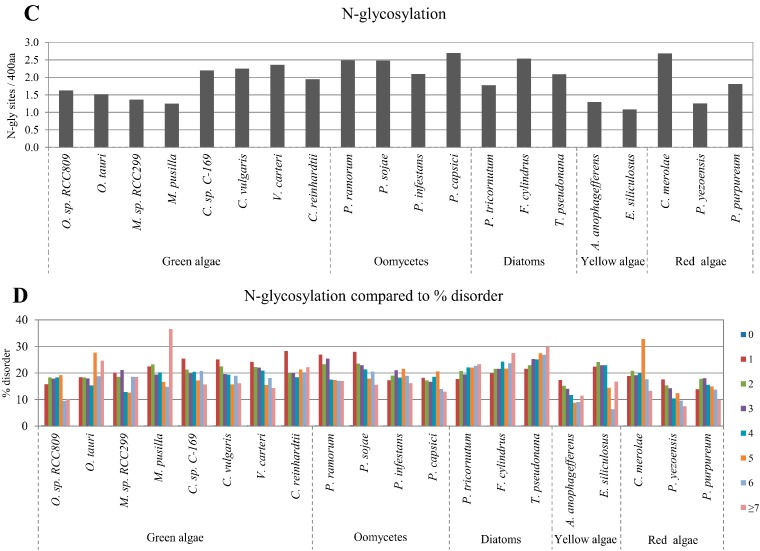
Correlation between disordered protein content and glycosylation sites. Normalized predicted O-glycosylation and N-glycosylation sites per 400 amino acids in the studied algae proteomes are presented in (**A**,**C**), respectively; The relative percentage of disordered protein content with different numbers of predicted O- and N-glycosylation sites are presented in (**B**,**D**), respectively.

Protein glycosylation is one of the most common PTMs where the attachment of glycans to specific residues affects protein folding, localization, and stability. Glycosylated proteins play an important role in essential biological functions such as immunogenicity, catalytic activity, viral clearance, and ligand-receptor interactions [34,35,36,37]. Two major types of protein glycosylation exist: O-linked and N-linked. O-glycosylation occurs frequently in eukaryotic cells and is involved in many basic cellular functions. However, significant differences in O-glycosylation patterns between plants and animals have been described, including the sites of glycan addition and glycan composition. O-glycosylation in plants mainly occurs on hydroxylproline (Hyp) residues [38]. The freely available NetOGlyc tool can be used to predict O-glycosylation sites in mammalian proteins [39]. However, there is currently no available prediction tool for O-glycosylation sites for plant proteins. Therefore, in the present study, we designed an original program to predict Hyp-O-linked glycosylation sites utilizing the consensus sequence for Hyp-O-linked glycosylation previously reported for plants [40]. The average abundance of O-glycosylations sites varied in the studied algae proteomes from approximately 0.8 to more than 1.7 sites per protein (Figure 3A). Additionally, we observed that the disordered protein content was positively correlated with the number of O-glycosylation sites (from 0 to ≥7) in every algae proteome analyzed (Figure 3B). The correlation between IDRs and O-glycosylation had high correlation coefficients and were found to be statistically significant (Table 1). The positive correlation between disordered protein content and the number of O-glycosylation sites was confirmed by using the alternative disorder prediction tool, RONN (Figure S4A).

Another major type of glycosylation, N-linked glycosylation, is arguably the most conserved form of protein glycosylation in eukaryotes [41]. As in other eukaryotic cells, N-linked glycosylation occurs frequently in plant cells and it has been shown that eukaryotic N-glycoproteins have invariant sequence recognition patterns, structural constraints, and subcellular localization [42]. N-linked glycosylation occurs on many secreted and membrane-bound glycoproteins as a co-translational process through the attachment to an asparagine (N) at the consensus motif asparagine-X-serine/threonine (NXS/T), in which X is any amino acid except proline [43]. For N-glycosylation site prediction, we used the prediction tool NetNglyc1.0 [44], which is noted to predict N-glycosylation sites in human proteins. However, the N-linked glycosylation pathway in algae shares a high degree of homology with that in other eukaryotic organisms [40,45]. Therefore we predicted N-linked glycosylation sites in the 20 algae proteomes by combining the results of the NetNglyc1.0 algorithm, and the existence of signal peptides by SignalP [46] and transmembrane regions by TMHMM [47] (Figure 3C). N-glycosylation correlated negatively with the disordered protein content in most of the studied algae proteomes. However, the results of the diatoms showed positive correlations between disordered protein content and N-glycosylation (Figure 3D, Table 1). These results were confirmed using the alternative disorder prediction tool, RONN (Figure S4B).

### 2.4. Correlation between Intrinsic Disorder and Ubiquitination and PEST (Region Enriched in Proline (P), Glutamic Acid (E), Serine (S), and Threonine (T) Residues)

**Figure 4:**
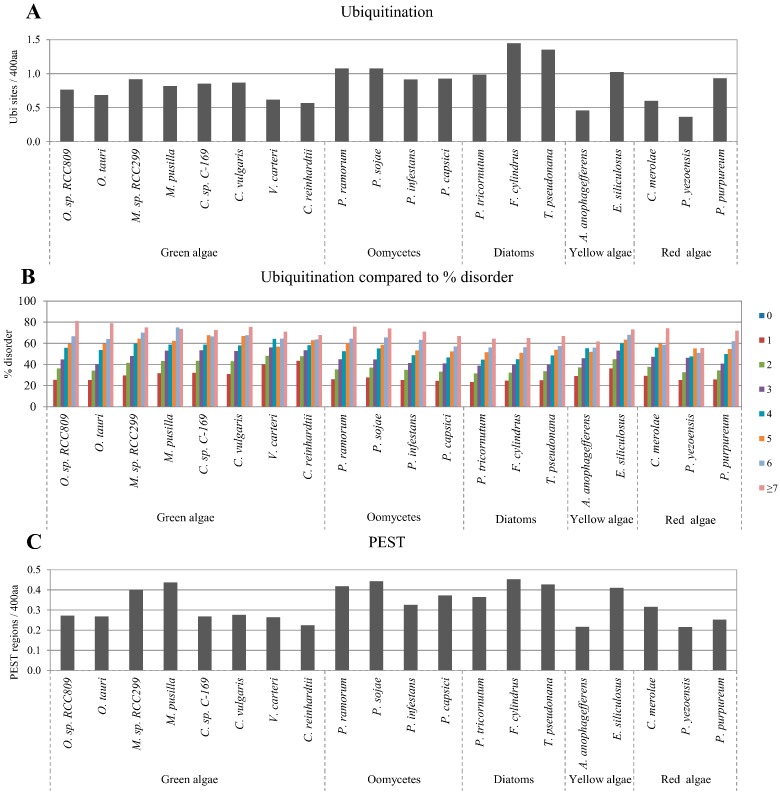

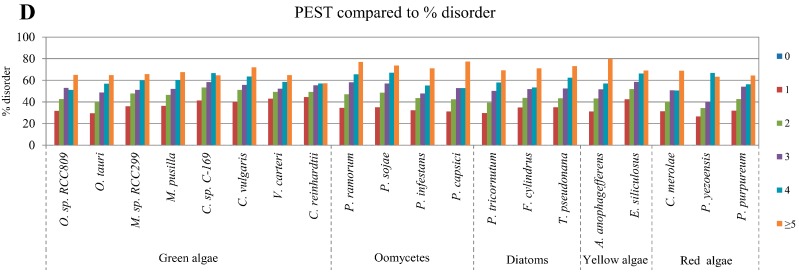
Correlation between disordered protein content and ubiquitination sites or PEST regions. Normalized predicted ubiquitination sites and PEST regions per 400 amino acids in the studied algae proteomes are presented in (**A**,**C**), respectively; Relative percentage of disordered protein content with different numbers of predicted sites of ubiquitination and predicted regions of PEST are presented in (**B**,**D**), respectively.

Ubiquitination sites and PEST regions within a protein sequence are related to protein degradation [48]. We used the freely available UbPred tool for ubiquitination site prediction and epestfind tool for PEST region prediction [49,50]. The content of ubiquitination sites varied from approximately 0.4 to 1.4 sites per 400 amino acids, and the content of PEST regions varied from approximately 0.2 to 0.5 sites per 400 amino acids in the analyzed algae proteomes (Figure 4A,C). We observed that the predicted presence of both ubiquitination sites and PEST regions were statistically positively correlated to disordered protein content in all algae proteomes (Figure 4B,D) with high correlation coefficients (Table 1). The observed correlations of ubiquitination and PEST with protein disorder were confirmed with the alternative disorder prediction tool, RONN (Figure S5).

### 2.5. Correlation of Intrinsic Disorder with Transmembrane Helices

**Figure 5:**
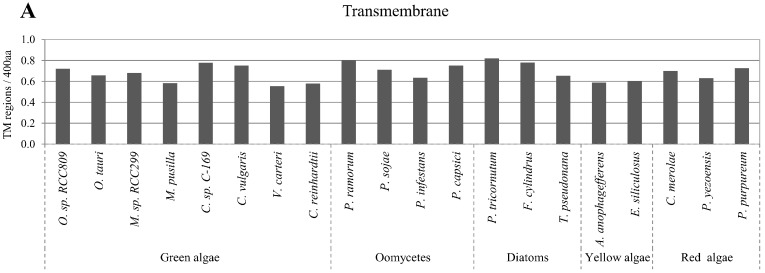

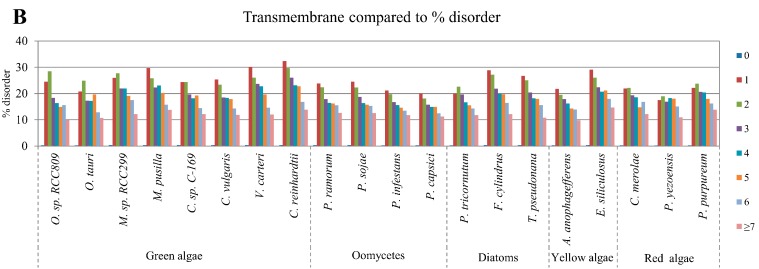
Correlation between disordered protein content and transmembrane regions. Normalized predicted transmembrane helices per 400 amino acids in the studied algae proteomes are presented in (**A**); Relative percentage of disordered protein content with different numbers of predicted transmembrane helices are presented in (**B**).

Transmembrane helices in proteins play an important role in the transport of various substances across biological membranes. In the present study, we used the freely available TMHMM tool for transmembrane helix prediction. The content of transmembrane helices varied in the analyzed algae proteomes from approximately 0.6 to 0.8 regions per 400 amino acids (Figure 5A). A negative correlation was observed between the number of predicted transmembrane helices and disordered protein content in all algae proteomes (Figure 5B). The observed correlations of the number of transmembrane helices with disordered protein content were confirmed with the alternative disorder prediction tool, RONN (Figure S6).

### 2.6. Relative Content of PTMs (Post-Translational Modifications) in Ordered and Disordered Regions of Algae Proteins

To further analyze the correlation between PTM sites and disordered regions, we determined the relative abundance of specific PTM sites including those for phosphorylation, O-glycosylation, and ubiquitination, in ordered or disordered regions of the algae proteomes. In accordance with a previous analysis [21], we used the relative abundance values for analysis, which are presented as a ratio of normalized PTM content in disordered and ordered segments (Rd/o) of algae proteomes (for details, see Experimental Section 4.3). This approach allowed us to estimate the robustness of the observed correlations between PTM sites and disordered regions. This analysis focused on site-specific PTMs rather than region-specific PTMs such as PEST and transmembrane helix content. Higher values of the Rd/o parameter were obtained for S- and T-phosphorylation as well as ubiquitination in the algae proteomes as a whole (18.8, 10.5, and 12.5, respectively). In contrast, the Rd/o parameter for Y-phosphorylation and N-glycosylation in total algae proteomes (1.3 and 1.2, respectively) indicated less robust relationships (Table 2). Incidentally, we found that oomycetes had the strongest correlation between phosphorylation and protein disorder compared to those of the other algae such as green algae, diatom, yellow algae, and red algae (Table 2).

**Table 2:** Relative post-translational modification (PTM) content in ordered and disordered regions of algae proteomes.

Category	S-pho	T-pho	Y-pho	O-gly	N-gly	Ubi
Green algae	15.1	8.4	1.1	3.2	0.9	11.0
Oomycetes	25.9	14.5	1.7	3.6	1.2	16.0
Diatom	19.8	10.3	1.3	2.9	1.9	11.9
Yellow algae	17.4	9.9	1.0	3.4	1.2	10.5
Red algae	19.2	11.3	1.6	2.6	1.2	16.6
Total	18.8	10.5	1.3	3.3	1.2	12.5

Ratios of normalized PTM contents (Rd/o) were calculated as described in the Experimental Section 4.3.

### 2.7. Relative Disordered Protein Content and the Number of PTMs (Post-Translational Modifications) in Specific and Common Protein Clusters of Algae Proteomes

In a previous study, protein disordered regions were associated with higher amino acids substitution rates [51]. Therefore, disordered proteins may evolve faster and hence are less conserved than structured proteins [52,53]. As such, there should be differences in the disordered protein content and the number of PTMs between species-specific protein clusters and common protein clusters of algae proteomes. For these calculations, we used the OrthoMCL tool to classify common protein clusters involving all 20 algae species used in this study, and species-specific protein clusters involving just one algae species. In total, we analyzed 15,205 common protein clusters and 66,858 species-specific protein clusters. The content of each PTM parameter such as protein disorder, phosphorylation, glycosylation, and ubiquitination in species-specific protein clusters or in common protein clusters are shown in Table 3. The content of PTM parameters was 1.4 to 3.4 times higher in species-specific protein clusters than in common protein clusters, as expected. Notably, in green algae, diatom, and yellow algae, the occurrence of S/T-phosphorylation in species-specific protein clusters was more than three times higher than in common protein clusters (Table S2).

**Table 3:** Preference of protein disorder and PTM in species-specific protein clusters and common protein clusters of algae proteomes.

Category	Disorder	S-pho	T-pho	Y-pho	O-gly	N-gly	Ubi
Specific	34%	2.1	1.0	0.7	1.4	2.1	1.0
Common	20%	0.6	0.3	0.5	0.8	1.3	0.6
Ratio of s/c	1.7	3.4	3.2	1.4	1.8	1.6	1.8

Total values of each parameter in species-specific protein clusters and common protein clusters are presented. The one-tailed *p* values as determined with *t*-tests between species-specific protein clusters and common protein clusters were less than 0.05. Ratio of s/c = the value of specific/the value of common.

## 3. Discussion

It was previously reported that disordered protein content was dependent on the length of the protein [54], while at the same time, disordered protein content is independent of proteome size [22]. In the current study, the degree of protein disorder was not associated with algae proteome size (Figure 1B), which was similar to a previous analysis [21].

Sequence redundancies before and after filtering were examined for every algae proteome (Figure S2), which were calculated as the number of removed sequences after filtering (subtracting the number of redundant sequences after filtering from that of before filtering) divided by the number before filtering. The numbers of sequences before and after filtering are given in Table S1. A strong positive correlation was observed between the content of redundant sequences and proteome size similar to previous results [21]. Additionally, it was found that the redundancy of amino acid sequences in the algae proteomes, which was approximately 1% to 27% (Figure S2), was lower as a whole than that in higher plants, which has been reported to be approximately 7% to 37% [21]. This implies that gene duplication occurs more often in higher plants than in algae, and that it is possible to obtain inaccurate results when sequences are not filtered for redundancy.

In the current study, we systematically investigated disordered protein content, sites of multiple PTMs, and regions of PEST and transmembrane helices in algae proteomes. In addition, we investigated the correlation between disordered protein content and the number of PTM sites or regions of PEST and transmembrane helices in algae proteomes. Previous studies have reported on the content of protein disorder in many species. For example, in prokaryotes, varying disordered protein content in different thermal groups has been observed [55]. However, disordered protein content is much higher in eukaryotes than in prokaryotes [2,56,57]. In higher plants, disordered protein content has been reported to be higher in monocot proteomes than that in dicot proteomes [21]. However, in the algae proteome analysis herein, a trend in disordered protein content among the algae groups such as green algae, oomycetes, diatom, yellow algae, and red algae was not found. This may be due to the inclusion of algae from a wide range of groups. The disordered protein content ranged from approximately 20% to 35% (Figure 1A). Thus, a more detailed comparison of integral algae characteristics should be performed to understand the differences of disordered protein content. A previous study has suggested that surface accessibility of enzyme-mediated reversible PTMs is closely related to protein-protein interactions in disordered regions [58]. Accordingly, we found that disordered protein content positively correlated with the presence of predicted PTM sites in the algae proteomes (Table 2). Specifically, there were strong correlations between disordered protein content and Ser- and Thr-phosphorylation, O-glycosylation, ubiquitination, and PEST regions (Table 1). Prior human and plant proteome-wide analyses have revealed that phosphorylation sites are significantly enriched in disordered proteins [16,19,23]. Thus, the results of our proteome-wide analysis of algae are similar to those of other eukaryotic proteome-wide analysis. This suggests that the present algae analysis can be applied to other eukaryotic data sets to determine common features of protein disorder and PTMs. No trend was observed for the average abundance of phosphorylation sites between algae groups (Figure 2A). However, a positive correlation between the predicted number (from 0 to ≥7) of Ser, Thr, and Tyr phosphorylation sites and disordered protein content was observed for every algae proteome (Figure 2B–D). These results are in agreement with past analyses of higher plant species [21]. Therefore, we suggest that the abundance of phosphorylation in disordered proteins is common in various algae and land plants.

We found that there was a low correlation between disordered protein content and Y-phosphorylation in algae, especially in yellow algae (Table 1). Similarly, small ratio values for Y-phosphorylation are presented in Table 2. Many PTMs including phosphorylation have been shown to occur in disordered regions, because IDRs are easily accessible and flexible. However, a recent study demonstrated that Y-phosphorylation does not tend to occur in disordered regions, which is in contrast to S- and T-phosphorylation [59]. Similarly, we found that Y-phosphorylation was predicted to occur preferentially in ordered regions.

It has previously been reported that the O-linked glycosylation sites are predominantly located in the IDRs of many eukaryotic proteins such those in as *Homo sapiens*, *Drosophila melanogaster*, *Caenorhabditis elegans*, *A. thaliana*, *Oryza sativa*, and *Schizosaccharomyces pombe* [5,16]. In the present study, a strong positive correlation between protein disorder and O-glycosylation was also observed in algae (Figure 3B, Table 1 and Table 2), which is similar to a previous report [21]. The positive correlation between protein disorder and O-glycosylation was expected because O-glycosylation in plants occurs on the Pro in consensus sequences [A/S/T/V]-P(1,4)-X(0,10)-[A/S/T/V]-P(1,4) [40], in which amino acids tend to form disorder regions [1,4,5,6,7,8]. Hence, the positive correlation between protein disorder and O-glycosylation can be considered as a universal trend in eukaryotes, including algae. In contrast to O-glycosylation, it is known that N-glycosylation does not strongly correlate with IDR content because N-glycosylation generally occurs co-translationally before a protein is fully folded. As a result, there is a lack of structural preference for N-glycosylation. Accordingly, no clear structural preference has been reported for N-glycosylation in proteins [29,60]. The correlation analysis in this study also failed to reveal a strong association between N-glycosylation and protein disorder in algae (Figure 3D, Table 1 and Table 2). However, the analysis results of the diatoms showed positive correlations between disordered protein content and N-glycosylation. (Figure 3D, Table 1). N-glycosylation is thought to be related to folding and stability of proteins, the extracellular matrix and cell-adhesion molecules [61]. In addition, most diatoms exude polymers from a slit or apical pore field in the siliceous cell wall. The exuded polymers consist of extracellular matrices, and are assembled into a variety of structures, such as trails (material left behind during motility), sheaths (organic matrices tightly associated with the cell wall), capsules (organic matrices loosely associated with the cell walls), and stalks (permanent attachment structures) [62]. Therefore, the result of correlation analysis between N-glycosylation and protein disorder implies that the living environment and lifestyle of diatoms may be related to N-glycosylation and protein disorder.

Ubiquitination is well known to be involved in protein degradation in eukaryotic cells. Similarly, PEST sequences represent a universal target for proteolytic degradation [48,63]. In several studies, IDR-related ubiquitination sites and regions of PEST within IDRs have been identified [4,64,65]. Therefore, we investigated the correlation between IDRs and the abundance of sequence motifs for ubiquitination and PEST regions. A strong positive correlation was observed between protein disorder and the predicted presence of both ubiquitination sites and PEST regions in all algae proteomes (Figure 4B,D and Table 1). These results provide further evidence that ubiquitination sites and PEST motifs preferentially occur in the flexible disordered regions of proteins including those in algae.

Transmembrane helices in proteins are important in a variety of critical and diverse biological processes. It has been reported that disordered protein content and the abundance of transmembrane helices has an influence on protein expression and solubility [4,64,65]. Therefore, we investigated the relation between the IDR content and the presence of transmembrane helices. A strong negative correlation was observed between the predicted presence of transmembrane helices and protein disorder in all algae proteomes (Figure 5B). This result was expected because transmembrane helices commonly consist of hydrophobic residues, whereas disordered regions primarily contain hydrophilic residues. Thus, the function of transmembrane helices is associated with structural stability rather than flexibility.

To further analyze correlations between PTMs sites and IDRs, we determined the relative abundances of site-specific PTMs sites including phosphorylation, O-glycosylation, and ubiquitination in ordered regions and disordered regions of algae proteomes. As a result, we observed that most values of Rd/o are over 1.0 indicating that PTMs primarily occur in the disordered regions (Table 2). However, Y-phosphorylation and N-glycosylation did not have a preference for disordered regions whereas S- and T-phosphorylation and ubiquitination had a strong preference for disordered regions.

Previous studies have reported that disordered regions are associated with higher amino acids substitution rates [51]. By comparing protein-protein interactions that occur in humans, flies, and yeast, it was found that interactions in disordered regions were significantly less conserved than in ordered regions [52,53]. Additionally, while evolutionarily conserved sequences within disordered regions are important for function, these conserved sequences account for only 5% of the disordered regions [66]. Therefore, disordered regions are less conserved than structured regions. In the present study, the association between protein disorder and PTM sites was higher in species-specific protein clusters than in common protein clusters in the algae proteomes as a whole (Table 3). Taken together, these results indicate that disordered regions, PTMs sites, and species-specific protein sequences are significantly related in algae proteomes.

## 4. Experimental Section

### 4.1. Data Sets

We used the following proteome sequence sets: for green algae, *Ostreococcus* sp. RCC809 (*O.* sp. RCC809) [67], *Ostreococcus* *tauri* (*O.* *tauri*) [67], *Micromonas* sp. RCC299 (*M.* sp. RCC299) [68], *Micromonas* *pusilla* CCMP1545 (*M.* *pusilla*) [68], *Coccomyxa* sp. C-169 (*C.* sp. C-169) [69], *Chlorella* *vulgaris* (*C.* *vulgaris*) [70], *Volvox* *carteri* (*V.* *carteri*) [71], and *Chlamydomonas* *reinhardtii* (*C.* *reinhardtii*) [72]; for oomycetes, *Phytophthora* *ramorum* (*P.* *ramorum*) [73], *Phytophthora* *sojae* (*P.* *sojae*) [73], *Phytophthora* *infestans* T30-4 (*P.* *infestans*) [74], and *Phytophthora* *capsici* (*P.* *capsici*) [75]; for diatom, *Phaeodactylum* *tricornutum* CCAP1055/1 (*P.* *tricornutum*) [76], *Fragilariopsis* *cylindrus* CCMP1102 (*F.* *cylindrus*), and *Thalassiosira* *pseudonana* CCMP1335 (*T. pseudonana*) [77]; for yellow algae, *Aureococcus* *anophagefferens* (*A.* *anophagefferens*) [78] and *Ectocarpus* *siliculosus* (*E.* *siliculosus*) [79]; and for red algae, *Cyanidioschyzon* *merolae* (*C.* *merolae*) [80], *Pyropia yezoensis* (*P. yezoensis*) [81], and *Porphyridium purpureum* (*P. purpureum*) [82].

The proteome sequences of *M.* sp. RCC299, *M.* *pusilla*, *C.* sp. C-169, and *V.* *carteri* were retrieved from Phytozome (http://www.phytozome.net) [83]. *O.* sp. RCC809, *O.* *tauri*, *C.* *vulgaris*, *C.* *reinhardtii*, *P.* *ramorum*, *P.* *sojae*, *P.* *infestans*, *P.* *capsici*, *P.* *tricornutum*, *F.* *cylindrus*, *T. pseudonana*, *A.* *anophagefferens*, *E.* *siliculosus*, *P. yezoensis*, and *P. purpureum* were retrieved from the Genome Portal of the Department of Energy Joint Genome Institute (http://genome.jgi-psf.org) [84]. *C.* *merolae* proteome sequence was obtained from the *Cyanidioschyzon merolae* Genome Project (http://merolae.biol.s.u-tokyo.ac.jp) [80]. Subsequently, we prepared non-redundant protein sequence sets. First, to construct the protein sequence datasets, amino acid sequences of less than 50 and more than 4000 amino acids in length were filtered out for impartial analysis. Next, we made protein clusters calculated with the OrthoMCL tool (version 1.4) [30] for the above 20 algae whole protein sequence sets. The threshold options of sequence alignments in OrthoMCL are “pi_cut off = 90%”, “pmatch_cut off = 90%”, and “pv_cut off = 1 × 10^−30^”. Finally, we obtained non-redundant sequence sets from the clustering result of OrthoMCL to extract representative sequences of each cluster and singlet sequences.

### 4.2. Prediction of Multiple Properties of Protein Sequences

We used prediction tools for multiple calculations on the proteome sequences of the above 20 algae. To calculate intrinsic disorder in proteins, DISOPRED (version 2.4.2) [31] and RONN (version 3) [32] were employed. The disordered protein content of each protein was obtained by dividing the number of disordered amino acid residues by the protein length. We analyzed the sites of Ser, Thr, and Tyr phosphorylation, N-linked Asn glycosylation, O-linked Pro glycosylation, and ubiquitination. Moreover, the regions of transmembrane helix domains and PEST regions were investigated. These PTMs and regions were predicted using the following bioinformatics algorithm or tools. Sites of phosphorylation were predicted with Musite (version 1.0.1) [17] downloaded from http://musite.sourceforge.net, which was used to predict phosphorylation sites in the target algae proteomes with “Eukaryote-General-Ser-Thr; Eukaryote-General-Tyr” rather than with “*A. thaliana*-General-Ser-Thr” as a prediction model. This was because oomycetes, which are regarded as colorless algae [85], were included in the algae species set in this analysis and the *A. thaliana* model did not contain Tyr-phosphorylation information. Sites of O-glycosylation were predicted based on the previously reported consensus sequence [A/S/T/V]-P(1,4)-X(0,10)-[A/S/T/V]-P(1,4) [40,45] for plant proteins containing sites of O-linked Pro glycosylation. While there is currently no prediction tool to detect O-glycosylation sites in plants, we made an original Perl script utilizing the aforementioned consensus sequences. Sites of N-glycosylation were predicted with the NetNGlyc tool (version 1.0) [44]. The N-glycosylation sites were predicted by combining the results of the NetNglyc tool, and the existence of signal peptides by SignalP (version 4.0) [46] and transmembrane regions by TMHMM (version 2.0) [47]. Sites of ubiquitination were predicted with the UbPred tool [86] downloaded from http://ubpred.org. Sites predicted with medium confidence (score range 0.69 ≤ *s* ≤ 0.84) were considered valid ubiquitination sites from the UbPred prediction results. Transmembrane helix regions were predicted with TMHMM (version 2.0) [47], and PEST regions were predicted with the epestfind tool of EMBOSS [49,50]. We applied the TMHMM and epestfind tools with default runtime parameters. The numbers of predicted PTM sites or regions (transmembrane helix and PEST regions) in proteins were normalized to the uniform length of 400 amino acids, rather than per sequence, to account for the difference in the average protein lengths in the datasets.

### 4.3. Relative Content of PTMs in Ordered and Disordered Segments

The tendency of specific PTM sites to occur in disordered and ordered region of each algae proteome was calculated by using appropriate algorithms following described methods [21]. Specifically, in accordance with a past analysis, the relative abundance of a specific PTM in the ordered and disordered segments of algae proteomes was analyzed using the following ratio: Rd/o = Nd/Ld:No/Lo, where No is the total number of PTM sites in the ordered segment of a proteome, Lo is the length of the ordered proteome segment, Nd is the total number of PTM sites in the disordered segment of a proteome, and Ld is the length of the disordered proteome segment. By this definition, the Rd/o value equals 1 if the relative abundances of a PTM in the ordered and disordered regions are the same. Furthermore, a value of >1 indicates when a PTM preferentially occurs in disordered regions, and a value of <1 indicates if a PTM tends to occur in ordered regions.

### 4.4. Classification of Species-Specific Proteins and Common Proteins of Algae Proteomes

The protein sequences were analyzed using the OrthoMCL tool [30] to classify species-specific and common proteins among the 20 algae proteomes. Pairwise sequence similarities between all protein sequences were calculated with BLASTP using an *e*-value cutoff of 1 × 10^−5^. Using these results, protein clusters that were equivalent to orthologous groups were estimated using the Markov clustering algorithm employed in OrthoMCL with the default runtime parameters. In this study, a singlet and a cluster consisting of only one species were regarded as a species-specific protein. In contrast, a cluster which consisted of all 20 species, was regarded as a common protein.

## 5. Conclusions

This analysis is the first large-scale bioinformatics study of IDRs in algae proteomes, which are considered common ancestors of plants. Our findings regarding the correlation between disordered protein content and multiple PTM sites, PEST, and transmembrane helices in algae proteomes may be useful for investigations into the unsolved biological roles of these proteins, and for understanding algae evolution. Information resources are currently being developed for various plant species from comprehensive analyses such as those in genetics and metabolomics [87,88,89,90,91,92,93]. As such, we aim to perform an integrative analysis of protein properties combined with the above proteomic data, and expect to reveal relationships between proteomics and other biological features in the future.

## Supplementary Materials

Supplementary materials can be found at http://www.mdpi.com/1422-0067/16/08/19812/s1.

## References

[1] Dunker A.K., Lawson J.D., Brown C.J., Williams R.M., Romero P., Oh J.S., Oldfield C.J., Campen A.M., Ratliff C.R., Hipps K.W. (2001). Intrinsically disordered protein. J. Mol. Graph. Model..

[2] Pancsa R., Tompa P. (2012). Structural disorder in eukaryotes. PLoS ONE.

[3] Ward J.J., Sodhi J.S., McGuffin L.J., Buxton B.F., Jones D.T. (2004). Prediction and functional analysis of native disorder in proteins from the three kingdoms of life. J. Mol. Biol..

[4] Hansen J.C., Lu X., Ross E.D., Woody R.W. (2006). Intrinsic protein disorder, amino acid composition, and histone terminal domains. J. Biol. Chem..

[5] Nishikawa I., Nakajima Y., Ito M., Fukuchi S., Homma K., Nishikawa K. (2010). Computational prediction of O-linked glycosylation sites that preferentially map on intrinsically disordered regions of extracellular proteins. Int. J. Mol. Sci..

[6] Romero P., Obradovic Z., Li X.H., Garner E.C., Brown C.J., Dunker A.K. (2001). Sequence complexity of disordered protein. Proteins.

[7] Shimizu K., Hirose S., Noguchi T. (2007). POODLE-S: Web application for predicting protein disorder by using physicochemical features and reduced amino acid set of a position-specific scoring matrix. Bioinformatics.

[8] Uversky V.N., Gillespie J.R., Fink A.L. (2000). Why are “natively unfolded” proteins unstructured under physiologic conditions?. Proteins.

[9] Uversky V.N., Oldfield C.J., Midic U., Xie H.B., Xue B., Vucetic S., Iakoucheva L.M., Obradovic Z., Dunker A.K. (2009). Unfoldomics of human diseases: Linking protein intrinsic disorder with diseases. BMC Genom..

[10] Bandaru V., Cooper W., Wallace S.S., Doublie S. (2004). Overproduction, crystallization and preliminary crystallographic analysis of a novel human DNA-repair enzyme that recognizes oxidative DNA damage. Acta Crystallogr. Sect. D Biol. Crystallogr..

[11] He B., Wang K.J., Liu Y.L., Xue B., Uversky V.N., Dunker A.K. (2009). Predicting intrinsic disorder in proteins: An overview. Cell Res..

[12] Uversky V.N. (2011). Intrinsically disordered proteins from A to Z. Int. J. Biochem. Cell Biol..

[13] Dunker A.K., Brown C.J., Lawson J.D., Iakoucheva L.M., Obradovic Z. (2002). Intrinsic disorder and protein function. Biochemistry.

[14] van der Lee R., Buljan M., Lang B., Weatheritt R.J., Daughdrill G.W., Dunker A.K., Fuxreiter M., Gough J., Gsponer J., Jones D.T. (2014). Classification of intrinsically disordered regions and proteins. Chem. Rev..

[15] Karve T.M., Cheema A.K. (2011). Small changes huge impact: The role of protein posttranslational modifications in cellular homeostasis and disease. J. Amino Acids.

[16] Fukuchi S., Hosoda K., Homma K., Gojobori T., Nishikawa K. (2011). Binary classification of protein molecules into intrinsically disordered and ordered segments. BMC Struct. Biol..

[17] Gao J., Thelen J.J., Dunker A.K., Xu D. (2010). Musite, a tool for global prediction of general and kinase-specific phosphorylation sites. Mol. Cell. Proteom..

[18] Iakoucheva L.M., Radivojac P., Brown C.J., O’Connor T.R., Sikes J.G., Obradovic Z., Dunker A.K. (2004). The importance of intrinsic disorder for protein phosphorylation. Nucleic Acids Res..

[19] Yao Q., Gao J., Bollinger C., Thelen J.J., Xu D. (2012). Predicting and analyzing protein phosphorylation sites in plants using musite. Front. Plant Sci..

[20] Gao J., Xu D. (2012). Correlation between posttranslational modification and intrinsic disorder in protein. Pac. Symp. Biocomput..

[21] Kurotani A., Tokmakov A.A., Kuroda Y., Fukami Y., Shinozaki K., Sakurai T. (2014). Correlations between predicted protein disorder and post-translational modifications in plants. Bioinformatics.

[22] Xue B., Dunker A.K., Uversky V.N. (2012). Orderly order in protein intrinsic disorder distribution: Disorder in 3500 proteomes from viruses and the three domains of life. J. Biomol. Struct. Dyn..

[23] Pietrosemoli N., Garcia-Martin J.A., Solano R., Pazos F. (2013). Genome-wide analysis of protein disorder in arabidopsis thaliana: Implications for plant environmental adaptation. PLoS ONE.

[24] Umezawa T., Sugiyama N., Takahashi F., Anderson J.C., Ishihama Y., Peck S.C., Shinozaki K. (2013). Genetics and phosphoproteomics reveal a protein phosphorylation network in the abscisic acid signaling pathway in *Arabidopsis thaliana*. Sci. Signal..

[25] The Gene Ontology Consortium (2015). Gene Ontology Consortium: Going forward. Nucleic Acids Res..

[26] Li D., Berardini T.Z., Muller R.J., Huala E. (2012). Building an efficient curation workflow for the *Arabidopsis* literature corpus. Database.

[27] Kourmpetis Y.A., van Dijk A.D., van Ham R.C., ter Braak C.J.F. (2011). Genome-wide computational function prediction of arabidopsis proteins by integration of multiple data sources. Plant Physiol..

[28] Akiyama K., Kurotani A., Iida K., Kuromori T., Shinozaki K., Sakurai T. (2013). RARGE II: An integrated phenotype database of *Arabidopsis* mutant traits using a controlled vocabulary. Plant Cell Physiol..

[29] Kurotani A., Yamada Y., Shinozaki K., Kuroda Y., Sakurai T. (2015). Plant-PrAS: A database of physicochemical and structural properties and novel functional regions in plant proteomes. Plant Cell Physiol..

[30] Chen F., Mackey A.J., Stoeckert C.J., Roos D.S. (2006). OrthoMCL-DB: Querying a comprehensive multi-species collection of ortholog groups. Nucleic Acids Res..

[31] Ward J.J., McGuffin L.J., Bryson K., Buxton B.F., Jones D.T. (2004). The disopred server for the prediction of protein disorder. Bioinformatics.

[32] Yang Z.R., Thomson R., McNeil P., Esnouf R.M. (2005). RONN: The bio-basis function neural network technique applied to the detection of natively disordered regions in proteins. Bioinformatics.

[33] Benjamini Y., Hochberg Y. (1995). Controlling the false discovery rate—A practical and powerful approach to multiple testing. J. Roy. Stat. Soc. B Met..

[34] Sanders S.L., Gentzsch M., Tanner W., Herskowitz I. (1999). O-glycosylation of Axl2/Bud10p by Pmt4p is required for its stability, localization, and function in daughter cells. J. Cell Biol.

[35] Narhi L.O., Arakawa T., Aoki K.H., Elmore R., Rohde M.F., Boone T., Strickland T.W. (1991). The effect of carbohydrate on the structure and stability of erythropoietin. J. Biol. Chem..

[36] Diaz C.L., Logman T.J.J., Stam H.C., Kijne J.W. (1995). Sugar-binding activity of pea lectin expressed in white clover hairy roots. Plant Physiol..

[37] Webster D.E., Thomas M.C. (2012). Post-translational modification of plant-made foreign proteins; glycosylation and beyond. Biotechnol. Adv..

[38] Nielsen K.K., Bojsen K., Roepstorff P., Mikkelsen J.D. (1994). A hydroxyproline-containing class-IV chitinase of sugar-beet is glycosylated with xylose. Plant Mol. Biol..

[39] Steentoft C., Vakhrushev S.Y., Joshi H.J., Kong Y., Vester-Christensen M.B., Schjoldager K.T.B.G., Lavrsen K., Dabelsteen S., Pedersen N.B., Marcos-Silva L. (2013). Precision mapping of the human *O*-GalNAc glycoproteome through simplecell technology. EMBO J..

[40] Gomord V., Fitchette A.C., Menu-Bouaouiche L., Saint-Jore-Dupas C., Plasson C., Michaud D., Faye L. (2010). Plant-specific glycosylation patterns in the context of therapeutic protein production. Plant Biotechnol. J..

[41] Wilson I.B.H. (2002). Glycosylation of proteins in plants and invertebrates. Curr. Opin. Struct. Biol..

[42] Lam P.V., Goldman R., Karagiannis K., Narsule T., Simonyan V., Soika V., Mazumder R. (2013). Structure-based comparative analysis and prediction of N-linked glycosylation sites in evolutionarily distant eukaryotes. Genom. Proteom. Bioinform..

[43] Stanley P., Schachter H., Taniguchi N., Varki A., Cummings R.D., Esko J.D., Freeze H.H., Stanley P., Bertozzi C.R., Hart G.W., Etzler M.E. (2009). N-glycans. Essentials of Glycobiology.

[44] Gupta R., Brunak S. (2002). Prediction of glycosylation across the human proteome and the correlation to protein function. Pac. Symp. Biocomput..

[45] Chauhan J.S., Rao A., Raghava G.P.S. (2013). *In silico* platform for prediction of N-, O- and C-glycosites in eukaryotic protein sequences. PLoS ONE.

[46] Petersen T.N., Brunak S., von Heijne G., Nielsen H. (2011). Signalp 4.0: Discriminating signal peptides from transmembrane regions. Nat. Methods.

[47] Krogh A., Larsson B., von Heijne G., Sonnhammer E.L. (2001). Predicting transmembrane protein topology with a hidden Markov model: Application to complete genomes. J. Mol. Biol..

[48] Rechsteiner M., Rogers S.W. (1996). Pest sequences and regulation by proteolysis. Trends Biochem. Sci..

[49] Rogers S., Wells R., Rechsteiner M. (1986). Amino-acid-sequences common to rapidly degraded proteins the PEST hypothesis. Science.

[50] Rice P., Longden I., Bleasby A. (2000). EMBOSS: The european molecular biology open software suite. Trends Genet..

[51] Brunquell J., Yuan J., Erwin A., Westerheide S.D., Xue B. (2014). DBC1/CCAR2 and CCAR1 are largely disordered proteins that have evolved from one common ancestor. Biomed. Res. Int..

[52] Kim P.M., Sboner A., Xia Y., Gerstein M. (2008). The role of disorder in interaction networks: A structural analysis. Mol. Syst. Biol..

[53] Mosca R., Pache R.A., Aloy P. (2012). The role of structural disorder in the rewiring of protein interactions through evolution. Mol. Cell. Proteom..

[54] Peng K., Radivojac P., Vucetic S., Dunker A.K., Obradovic Z. (2006). Length-dependent prediction of protein intrinsic disorder. BMC Bioinform..

[55] Burra P.V., Kalmar L., Tompa P. (2010). Reduction in structural disorder and functional complexity in the thermal adaptation of prokaryotes. PLoS ONE.

[56] Schad E., Tompa P., Hegyi H. (2011). The relationship between proteome size, structural disorder and organism complexity. Genome Biol..

[57] Oates M.E., Romero P., Ishida T., Ghalwash M., Mizianty M.J., Xue B., Dosztanyi Z., Uversky V.N., Obradovic Z., Kurgan L. (2013). D2P2: Database of disordered protein predictions. Nucleic Acids Res..

[58] Pang C.N.I., Hayen A., Wilkins M.R. (2007). Surface accessibility of protein post-translational modifications. J. Proteome Res..

[59] Sirota F.L., Maurer-Stroh S., Eisenhaber B., Eisenhaber F. (2015). Single-residue posttranslational modification sites at the N-terminus, C-terminus or in-between: To be or not to be exposed for enzyme access. Proteomics.

[60] Petrescu A.J., Milac A.L., Petrescu S.M., Dwek R.A., Wormald M.R. (2004). Statistical analysis of the protein environment of N-glycosylation sites: Implications for occupancy, structure, and folding. Glycobiology.

[61] Varki A., Esko J.D., Colley K.J., Varki A., Cummings R.D., Esko J.D., Freeze H.H., Stanley P., Bertozzi C.R., Hart G.W., Etzler M.E. (2009). Cellular organization of glycosylation. Essentials of Glycobiology.

[62] Wustman B.A., Gretz M.R., Hoagland K.D. (1997). Extracellular matrix assembly in diatoms (Bacillariophyceae). I. A model of adhesives based on chemical characterization and localization of polysaccharides from the marine diatom achnanthes longipes and other diatoms. Plant Physiol..

[63] Belizario J.E., Alves J., Garay-Malpartida M., Occhiucci J.M. (2008). Coupling caspase cleavage and proteasomal degradation of proteins carrying PEST motif. Curr. Protein Pept. Sci..

[64] Dunker A.K., Silman I., Uversky V.N., Sussman J.L. (2008). Function and structure of inherently disordered proteins. Curr. Opin. Struct. Biol..

[65] Tokmakov A.A., Kurotani A., Shirouzu M., Fukami Y., Yokoyama S. (2014). Bioinformatics analysis and optimization of cell-free protein synthesis. Methods Mol. Biol..

[66] Ba A.N.N., Yeh B.J., van Dyk D., Davidson A.R., Andrews B.J., Weiss E.L., Moses A.M. (2012). Proteome-wide discovery of evolutionary conserved sequences in disordered regions. Sci. Signal..

[67] Palenik B., Grimwood J., Aerts A., Rouze P., Salamov A., Putnam N., Dupont C., Jorgensen R., Derelle E., Rombauts S. (2007). The tiny eukaryote ostreococcus provides genomic insights into the paradox of plankton speciation. Proc. Natl. Acad. Sci. USA.

[68] Worden A.Z., Lee J.H., Mock T., Rouze P., Simmons M.P., Aerts A.L., Allen A.E., Cuvelier M.L., Derelle E., Everett M.V. (2009). Green evolution and dynamic adaptations revealed by genomes of the marine picoeukaryotes micromonas. Science.

[69] Blanc G., Agarkova I., Grimwood J., Kuo A., Brueggeman A., Dunigan D.D., Gurnon J., Ladunga I., Lindquist E., Lucas S. (2012). The genome of the polar eukaryotic microalga *Coccomyxa subellipsoidea* reveals traits of cold adaptation. Genome Biol..

[70] Wakasugi T., Nagai T., Kapoor M., Sugita M., Ito M., Ito S., Tsudzuki J., Nakashima K., Tsudzuki T., Suzuki Y. (1997). Complete nucleotide sequence of the chloroplast genome from the green alga chlorella vulgaris: The existence of genes possibly involved in chloroplast division. Proc. Natl. Acad. Sci. USA.

[71] Prochnik S.E., Umen J., Nedelcu A.M., Hallmann A., Miller S.M., Nishii I., Ferris P., Kuo A., Mitros T., Fritz-Laylin L.K. (2010). Genomic analysis of organismal complexity in the multicellular green alga *Volvox carteri*. Science.

[72] Merchant S.S., Prochnik S.E., Vallon O., Harris E.H., Karpowicz S.J., Witman G.B., Terry A., Salamov A., Fritz-Laylin L.K., Marechal-Drouard L. (2007). The chlamydomonas genome reveals the evolution of key animal and plant functions. Science.

[73] Tyler B.M., Tripathy S., Zhang X.M., Dehal P., Jiang R.H.Y., Aerts A., Arredondo F.D., Baxter L., Bensasson D., Beynon J.L. (2006). Phytophthora genome sequences uncover evolutionary origins and mechanisms of pathogenesis. Science.

[74] Haas B.J., Kamoun S., Zody M.C., Jiang R.H.Y., Handsaker R.E., Cano L.M., Grabherr M., Kodira C.D., Raffaele S., Torto-Alalibo T. (2009). Genome sequence and analysis of the irish potato famine pathogen *Phytophthora infestans*. Nature.

[75] Lamour K.H., Mudge J., Gobena D., Hurtado-Gonzales O.P., Schmutz J., Kuo A., Miller N.A., Rice B.J., Raffaele S., Cano L.M. (2012). Genome sequencing and mapping reveal loss of heterozygosity as a mechanism for rapid adaptation in the vegetable pathogen *Phytophthora capsici*. Mol. Plant Microbe Interact..

[76] Bowler C., Allen A.E., Badger J.H., Grimwood J., Jabbari K., Kuo A., Maheswari U., Martens C., Maumus F., Otillar R.P. (2008). The phaeodactylum genome reveals the evolutionary history of diatom genomes. Nature.

[77] Armbrust E.V., Berges J.A., Bowler C., Green B.R., Martinez D., Putnam N.H., Zhou S.G., Allen A.E., Apt K.E., Bechner M. (2004). The genome of the diatom *Thalassiosira pseudonana*: Ecology, evolution, and metabolism. Science.

[78] Gobler C.J., Berry D.L., Dyhrman S.T., Wilhelm S.W., Salamov A., Lobanov A.V., Zhang Y., Collier J.L., Wurch L.L., Kustka A.B. (2011). Niche of harmful alga *Aureococcus anophagefferens* revealed through ecogenomics. Proc. Natl. Acad. Sci. USA.

[79] Cock J.M., Sterck L., Rouze P., Scornet D., Allen A.E., Amoutzias G., Anthouard V., Artiguenave F., Aury J.M., Badger J.H. (2010). The ectocarpus genome and the independent evolution of multicellularity in brown algae. Nature.

[80] Matsuzaki M., Misumi O., Shin I.T., Maruyama S., Takahara M., Miyagishima S.Y., Mori T., Nishida K., Yagisawa F., Nishida K. (2004). Genome sequence of the ultrasmall unicellular red alga *Cyanidioschyzon merolae* 10D. Nature.

[81] Nakamura Y., Sasaki N., Kobayashi M., Ojima N., Yasuike M., Shigenobu Y., Satomi M., Fukuma Y., Shiwaku K., Tsujimoto A. (2013). The first symbiont-free genome sequence of marine red alga, Susabi-nori (*Pyropia yezoensis*). PLoS ONE.

[82] Bhattacharya D., Price D.C., Chan C.X., Qiu H., Rose N., Ball S., Weber A.P.M., Arias M.C., Henrissat B., Coutinho P.M. (2013). Genome of the red alga *Porphyridium purpureum*. Nat. Commun..

[83] Goodstein D.M., Shu S.Q., Howson R., Neupane R., Hayes R.D., Fazo J., Mitros T., Dirks W., Hellsten U., Putnam N. (2012). Phytozome: A comparative platform for green plant genomics. Nucleic Acids Res..

[84] Nordberg H., Cantor M., Dusheyko S., Hua S., Poliakov A., Shabalov I., Smirnova T., Grigoriev I.V., Dubchak I. (2014). The genome portal of the department of energy joint genome institute: 2014 updates. Nucleic Acids Res..

[85] Rossman A.Y., Palm M.E. (2006). Why are phytophthora and other oomycota not true fungi?. Outlooks Pest Manag..

[86] Radivojac P., Vacic V., Haynes C., Cocklin R.R., Mohan A., Heyen J.W., Goebl M.G., Iakoucheva L.M. (2010). Identification, analysis, and prediction of protein ubiquitination sites. Proteins.

[87] Cao J., Schneeberger K., Ossowski S., Gunther T., Bender S., Fitz J., Koenig D., Lanz C., Stegle O., Lippert C. (2011). Whole-genome sequencing of multiple *Arabidopsis thaliana* populations. Nat. Genet..

[88] Rafalski A. (2002). Applications of single nucleotide polymorphisms in crop genetics. Curr. Opin. Plant Biol..

[89] Sakurai T., Mochida K., Yoshida T., Akiyama K., Ishitani M., Seki M., Shinozaki K. (2013). Genome-wide discovery and information resource development of DNA polymorphisms in cassava. PLoS ONE.

[90] Bais P., Moon S.M., He K., Leitao R., Dreher K., Walk T., Sucaet Y., Barkan L., Wohlgemuth G., Roth M.R. (2010). PlantMetabolomics.Org: A web portal for plant metabolomics experiments. Plant Physiol..

[91] Akiyama K., Chikayama E., Yuasa H., Shimada Y., Tohge T., Shinozaki K., Hirai M., Sakurai T., Kikuchi J., Saito K. (2008). PRIMe: A web site that assembles tools for metabolomics and transcriptomics. In Silico Biol..

[92] Kudo T., Akiyama K., Kojima M., Makita N., Sakurai T., Sakakibara H. (2013). UniVIO: A multiple omics database with hormonome and transcriptome data from rice. Plant Cell Physiol..

[93] Sakurai T., Yamada T., Sawada Y., Matsuda F., Akiyama K., Shinozaki K., Hirai M.Y., Saito K. (2013). PRIMe update: Innovative content for plant metabolomics and integration of gene expression and metabolite accumulation. Plant Cell Physiol..

