# Supplementary material for: In Silico Analysis of Correlations between Protein Disorder and Post-Translational Modifications in Algae
Source: Int J Mol Sci. 2015 Aug 20;16(8):19812–35. doi: 10.3390/ijms160819812 (PMC4581327; doi:10.3390/ijms160819812)
Supplement: Supplementary file 1 [file ijms-16-19812-s001.zip › ijms-88641-Supplementary tables and figures-for publish.pdf]

Supplementary Information

Table S1. Total number and average length of protein sequences in the analyzed algae proteomes.

Species	Number of Sequences		Average Protein Sequence Length after Filtering
	Before Filtering	After Filtering	
O. sp. RCC809	7773	7670	378
O. tauri	7725	7606	383
M. sp. RCC299	10,103	9956	471
M. pusilla	10,660	10,542	440
C. sp. C-169	9629	9367	423
C. vulgaris	9994	9760	401
V. carteri	14,542	13,852	562
C. reinhardtii	16,706	15,624	493
P. ramorum	15,743	13,354	487
P. sojae	19,027	16,643	496
P. infestans	22,658	18,825	394
P. capsici	17,414	16,411	382
P. tricornutum	10,387	10,036	457
F. cylindrus	27,143	19,908	403
T. pseudonana	11,637	11,452	490
A. anophagefferens	11,501	10,754	507
E. siliculosus	16,533	15,318	516
C. merolae	5014	4855	503
P. yezoensis	10,327	9859	288
P. purpureum	8355	7955	463

Data filtering was performed as described in Experimental Section.

Table S2. Preferences of multiple PTMs for protein disorder in species-specific and common protein clusters of each algae proteomes.

Species			Disorder	S-pho	T-pho	Y-pho	O-gly	N-gly	Ubi
Green algae	*O.* sp. RCC809	specific	38%	3.1	2.1	0.8	1.2	2.2	1.2
		common	17%	0.4	0.3	0.5	0.6	1.7	0.5
		p-value	<0.05	<0.05	<0.05	<0.05	<0.05	0.222	<0.05
		ratio of s/c	2.2	7.1	8.0	1.6	2.0	1.3	2.2
	O. tauri	specific	36%	4.0	2.4	0.6	1.8	1.5	0.7
		common	18%	0.5	0.3	0.5	0.6	1.1	0.5
		p-value	<0.05	<0.05	<0.05	<0.05	<0.05	0.213	<0.05
		ratio of s/c	2.0	8.3	7.9	1.2	3.1	1.4	1.4
	M. sp. RCC299	specific	41%	3.4	1.9	0.8	1.9	1.6	1.2
		common	20%	0.6	0.4	0.5	0.8	1.0	0.6
		p-value	<0.05	<0.05	<0.05	<0.05	<0.05	0.095	<0.05
		ratio of s/c	2.0	5.4	5.0	1.6	2.5	1.6	2.0
	M. pusilla	specific	45%	3.2	1.7	0.7	2.1	1.2	1.0
		common	20%	0.5	0.3	0.5	0.8	1.4	0.6
		p-value	<0.05	<0.05	<0.05	<0.05	<0.05	0.309	<0.05
		ratio of s/c	2.2	6.2	6.1	1.4	2.6	0.8	1.8
	C. sp. C-169	specific	36%	2.0	0.9	0.6	1.6	1.6	1.1
		common	19%	0.5	0.3	0.5	0.8	1.1	0.6
		p-value	<0.05	<0.05	0.143	<0.05	<0.05	0.161	<0.05
		ratio of s/c	1.9	4.3	3.1	1.2	2.1	1.4	2.0
	C. vulgaris	specific	42%	2.3	1.0	0.6	1.7	1.9	1.1
		common	20%	0.5	0.3	0.5	0.8	1.3	0.6
		p-value	<0.05	0.054	0.111	<0.05	<0.05	0.108	<0.05
		ratio of s/c	2.2	4.6	3.2	1.2	2.1	1.4	2.0

Table S2. *Cont.*

Species			Disorder	S-pho	T-pho	Y-pho	O-gly	N-gly	Ubi
Green algae	*V. carteri*	specific	38%	2.3	1.1	0.7	1.9	2.3	0.7
		common	22%	0.6	0.3	0.5	0.9	1.2	0.5
		p-value	<0.05	<0.05	<0.05	<0.05	<0.05	<0.05	<0.05
		ratio of s/c	1.7	4.0	3.3	1.5	2.0	1.9	1.3
	C. reinhardtii	specific	40%	2.3	1.1	0.7	2.1	1.9	0.6
		common	23%	0.6	0.3	0.5	1.0	1.1	0.5
		p-value	<0.05	<0.05	<0.05	<0.05	<0.05	0.053	0.059
		ratio of s/c	1.7	4.2	3.4	1.4	2.1	1.7	1.1
Oomycetes	*P. ramorum*	specific	31%	1.7	1.0	0.6	1.1	1.9	1.1
		common	19%	0.6	0.3	0.5	0.7	1.6	0.7
		p-value	<0.05	<0.05	<0.05	<0.05	<0.05	<0.05	<0.05
		ratio of s/c	1.6	2.7	2.9	1.4	1.5	1.2	1.7
	P. sojae	specific	31%	1.9	1.0	0.6	1.2	2.2	1.0
		common	20%	0.7	0.4	0.5	0.8	1.2	0.7
		p-value	<0.05	<0.05	<0.05	<0.05	<0.05	0.201	<0.05
		ratio of s/c	1.5	2.7	2.9	1.4	1.6	1.9	1.5
	P. infestans	specific	23%	1.1	0.6	0.6	0.8	1.6	0.7
		common	20%	0.7	0.4	0.5	0.7	2.2	0.7
		p-value	<0.05	<0.05	<0.05	<0.05	<0.05	0.363	0.298
		ratio of s/c	1.2	1.6	1.6	1.4	1.2	0.7	1.0
	P. capsici	specific	23%	1.2	0.6	0.5	0.9	1.9	0.9
		common	18%	0.5	0.3	0.4	0.7	2.1	0.6
		p-value	<0.05	<0.05	<0.05	<0.05	<0.05	0.069	<0.05
		ratio of s/c	1.2	2.6	2.5	1.5	1.3	0.9	1.4

Table S2. *Cont.*

Species			Disorder	S-pho	T-pho	Y-pho	O-gly	N-gly	Ubi
Diatoms	*P. tricornutum*	specific	30%	2.2	1.1	0.7	1.4	2.5	1.5
		common	17%	0.5	0.2	0.5	0.6	1.0	0.6
		p-value	<0.05	<0.05	<0.05	<0.05	<0.05	<0.05	<0.05
		ratio of s/c	1.8	4.7	5.1	1.5	2.4	2.4	2.6
	F. cylindrus	specific	34%	1.8	0.9	0.7	0.9	3.1	1.7
		common	17%	0.4	0.2	0.4	0.5	1.3	0.8
		p-value	<0.05	<0.05	<0.05	<0.05	<0.05	<0.05	<0.05
		ratio of s/c	2.2	5.7	5.4	1.8	2.3	2.4	3.1
	T. pseudonana	specific	37%	2.5	1.1	0.9	1.3	2.5	2.0
		common	17%	0.4	0.2	0.5	0.5	1.1	0.6
		p-value	<0.05	<0.05	<0.05	<0.05	<0.05	<0.05	<0.05
		ratio of s/c	2.2	5.7	5.4	1.8	2.3	2.3	3.1
Yellow algae	*A. anophagefferens*	specific	27%	1.6	0.7	0.7	1.8	1.5	0.6
		common	16%	0.4	0.2	0.5	1.0	0.7	0.3
		p-value	<0.05	<0.05	<0.05	<0.05	<0.05	0.063	<0.05
		ratio of s/c	1.7	3.8	3.3	1.4	1.8	2.2	1.6
	E. siliculosus	specific	42%	3.1	1.5	0.6	1.4	1.2	1.2
		common	25%	1.2	0.5	0.5	0.9	0.9	0.7
		p-value	<0.05	<0.05	<0.05	<0.05	<0.05	<0.05	<0.05
		ratio of s/c	1.7	2.7	2.9	1.3	1.7	1.3	2.4

Table S2. *Cont.*

Species			Disorder	S-pho	T-pho	Y-pho	O-gly	N-gly	Ubi
Red algae	*C. merolae*	specific	29%	1.9	1.1	0.6	1.4	2.9	0.8
		common	22%	0.9	0.5	0.5	0.9	2.0	0.5
		p-value	<0.05	0.084	<0.05	<0.05	<0.05	0.301	<0.05
		ratio of s/c	1.3	2.1	2.2	1.3	1.6	1.4	1.5
	P. yezoensis	specific	21%	1.3	0.8	0.6	1.6	1.2	0.4
		common	19%	0.8	0.5	0.5	1.3	1.1	0.3
		p-value	<0.05	<0.05	<0.05	<0.05	<0.05	<0.05	<0.05
		ratio of s/c	1.1	1.6	1.5	1.2	1.2	1.1	1.6
	P. purpureum	specific	29%	1.9	0.8	0.6	1.1	2.0	1.3
		common	22%	0.7	0.3	0.5	0.7	1.1	0.7
		p-value	<0.05	0.342	0.053	<0.05	<0.05	0.081	<0.05
		ratio of s/c	1.3	2.6	2.5	1.1	1.4	1.8	1.8

Total values of each parameter, *p*-value from t-test and Ratio of s/c (the value of specific/the value of common) are indicated.

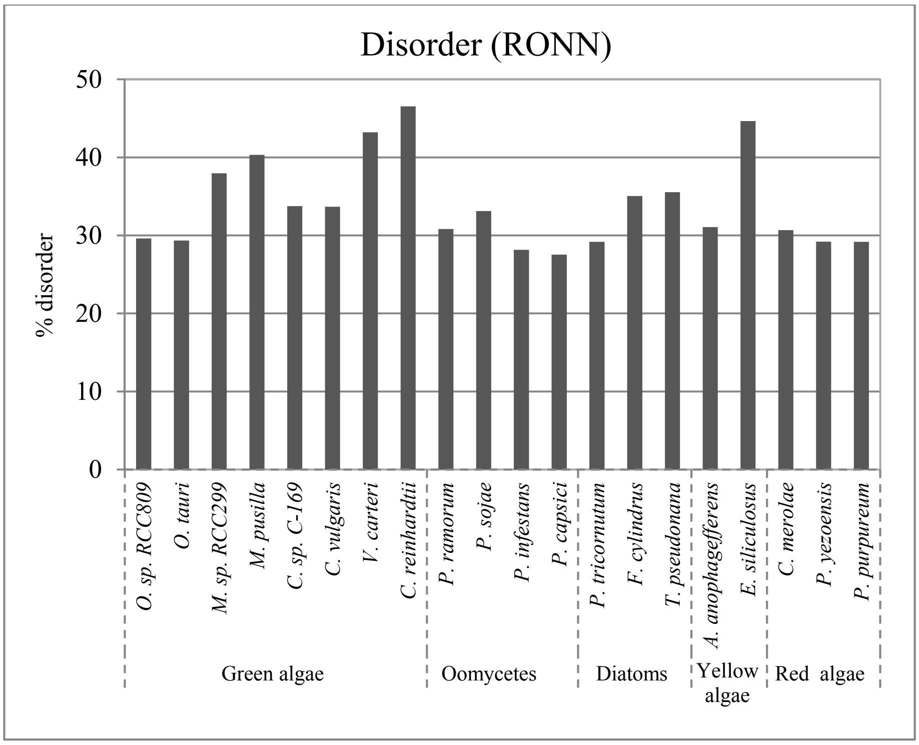

Figure S1. Predictive disordered protein content using the RONN tool in the studied algae proteomes.

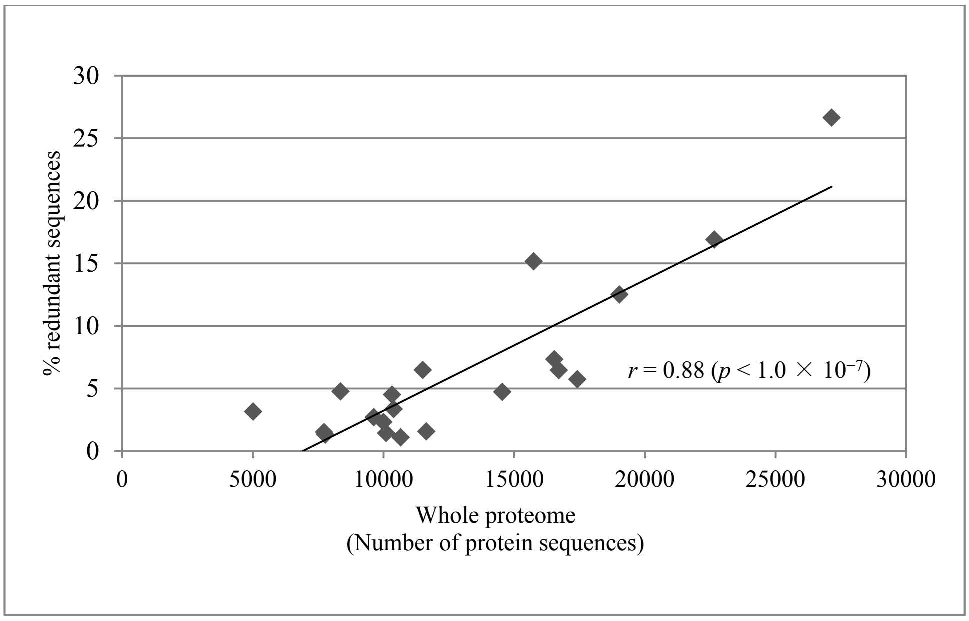

Figure S2. Correlation between protein redundancy and proteome size. The percentage of redundant sequences of each algal species was calculated as the number of removed sequences divided by the number of sequences before filtering sequence redundancy. The numbers of sequences before and after filtering sequence redundancy are shown in Table S1. The correlation coefficient and one-tailed probability values are indicated.

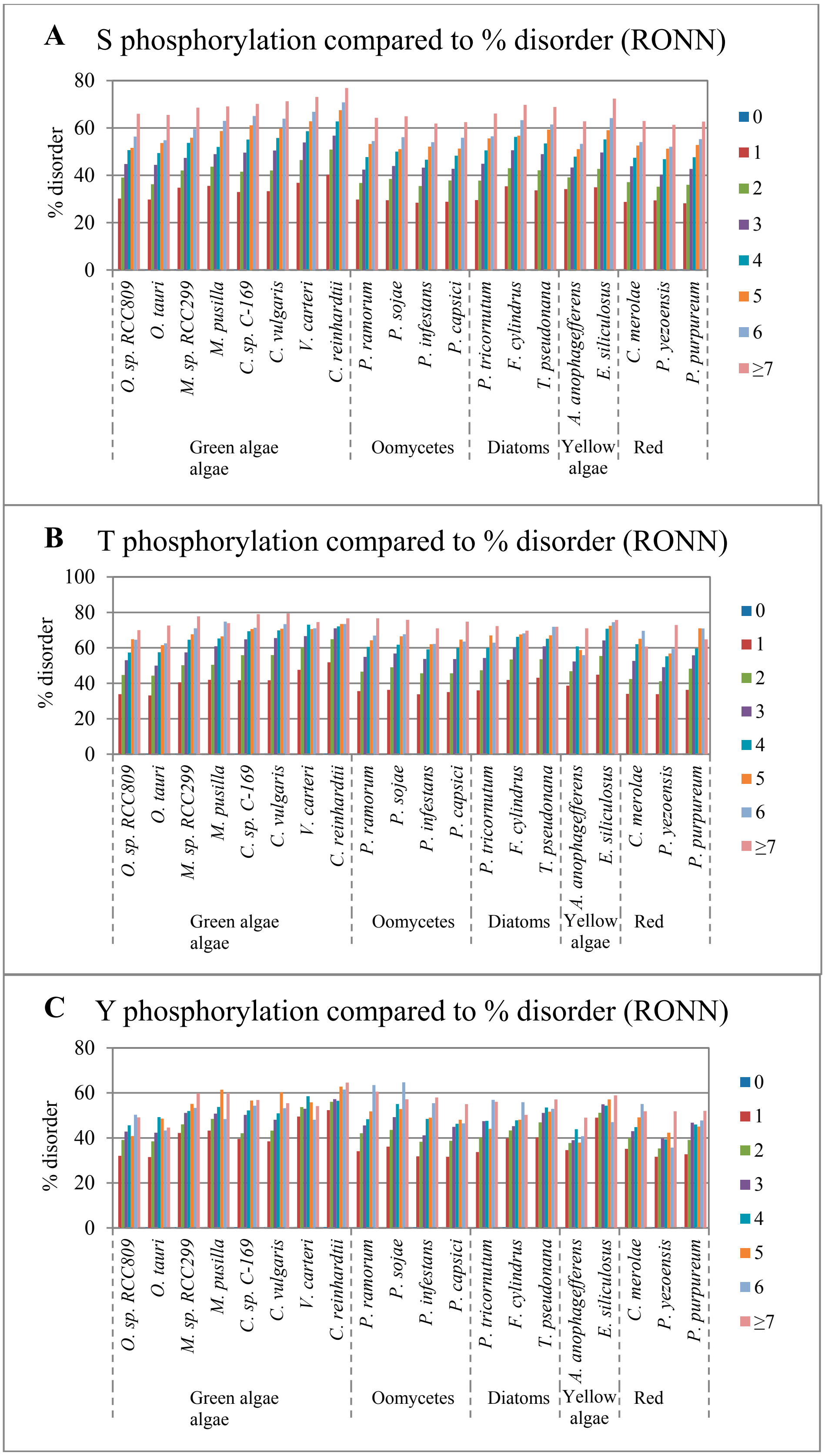

Figure S3. Correlation between disordered protein content and S/T/Y phosphorylation sites. Relative percentages of protein disorder calculated with the RONN tool with different numbers of predicted S, T, and Y phosphorylation sites, are presented in (**A**–**C**) respectively.

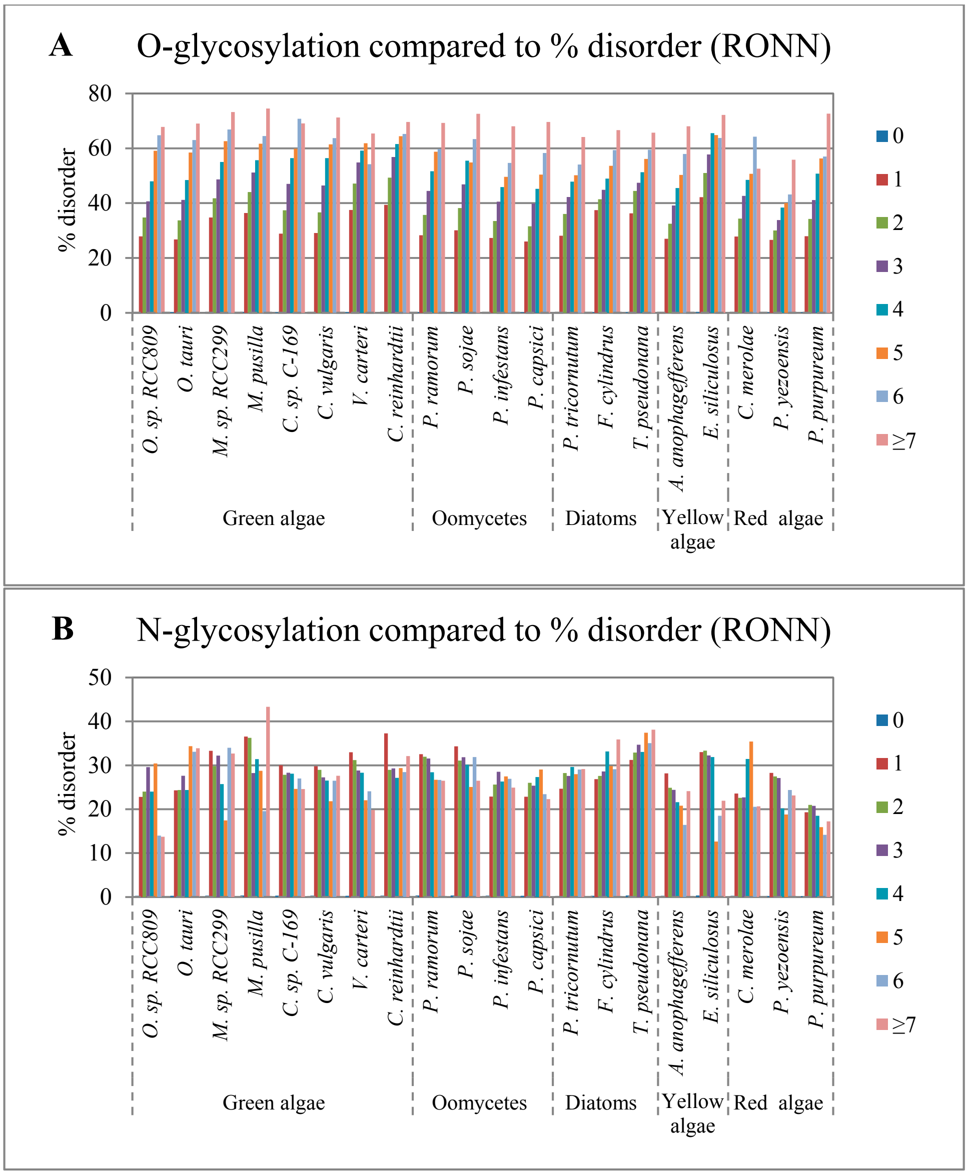

Figure S4. Correlation between disordered protein content and glycosylation. Relative percentages of protein disorder calculated with RONN tool with different numbers of predicted sites of O- and N-glycosylation, are presented in (**A**,**B**), respectively.

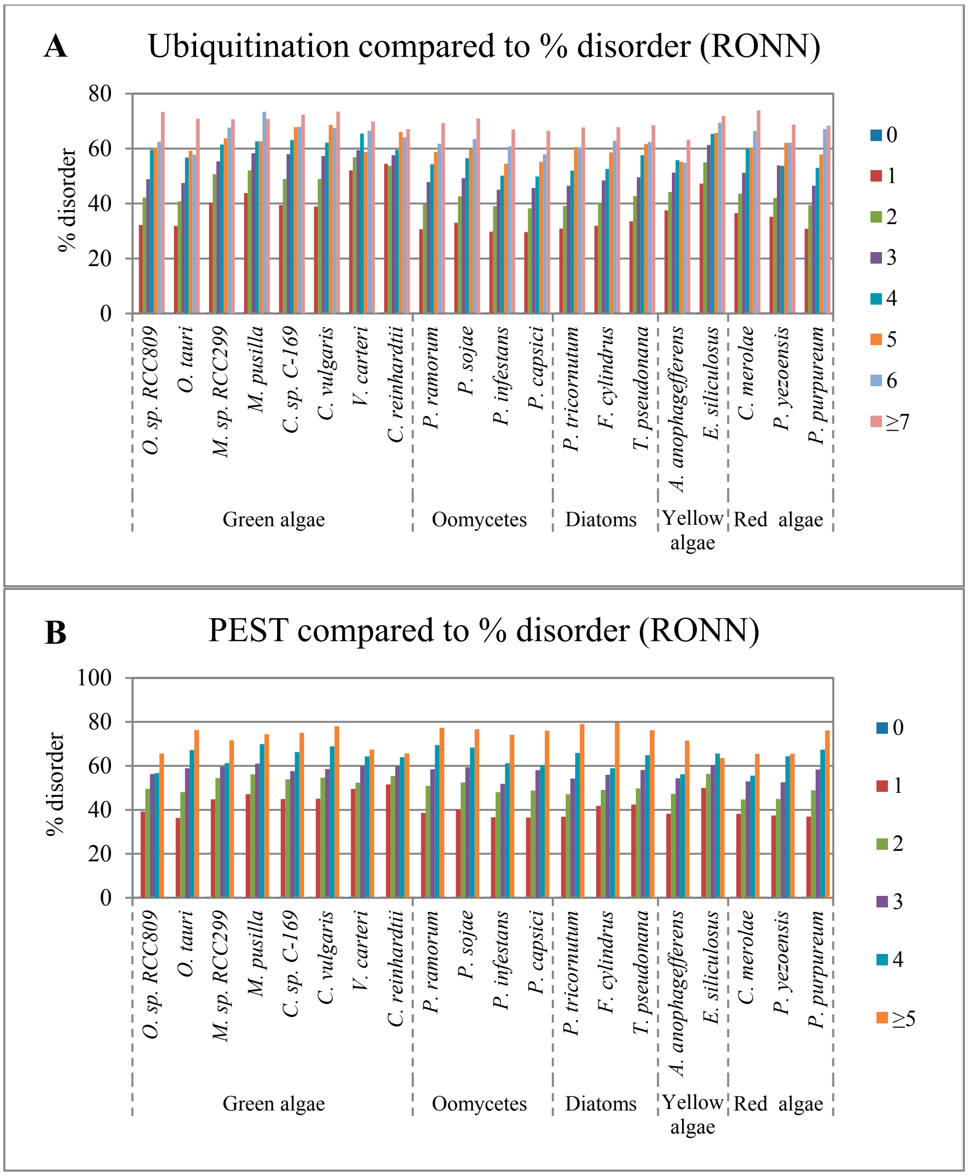

Figure S5. Correlations between disordered protein content and ubiquitination or PEST. Relative percentages of protein disorder calculated with RONN tool with different numbers of predicted sites of ubiquitination and predicted regions of PEST, are presented in (**A**,**B**), respectively.

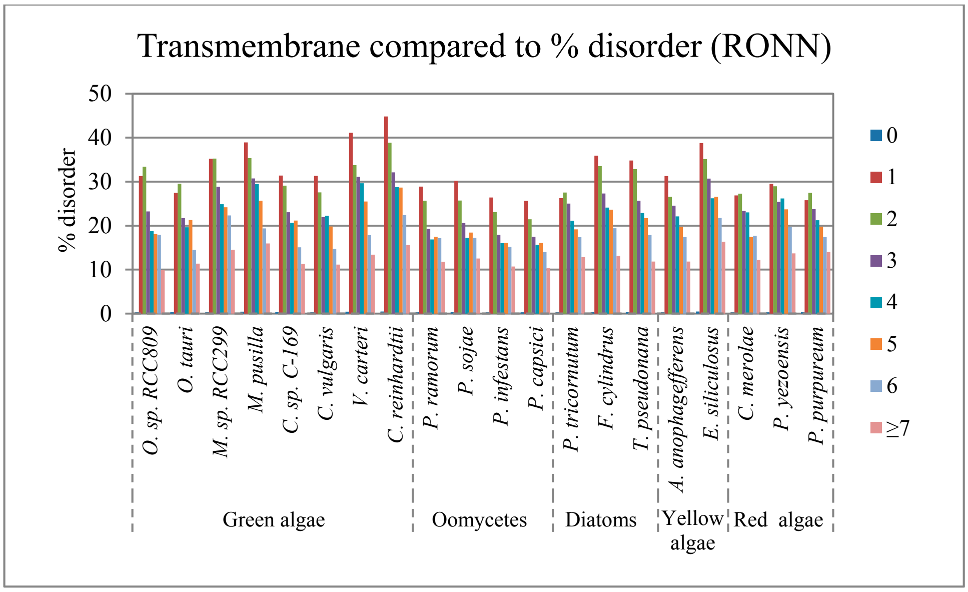

Figure S6. Correlations between disordered protein content and transmembrane helices. Relative percentages of protein disorder calculated with RONN tool with different numbers of predicted regions transmembrane helices, are presented.
